# Is Supplementation Efficacious in Maintaining Adequate Plasma Levels of Vitamin A and E for Thalassemic Patients Undergoing Hematopoietic Stem Cell Transplantation? A Cross-Sectional Study

**Published:** 2014-01-02

**Authors:** Mannan Hajimahmoodi, Molouk Hadjibabaie, Amir-Ali Hamidieh, Alireza Ahmadvand, Sahebeh Kazempanah, Naficeh Sadeghi, Ava Mansouri, Ardeshir Ghavamzadeh

**Affiliations:** 1 Department of Drug and Food Control; 2 Department of Clinical Pharmacy,Faculty of Pharmacy; 3 Research Center for Rational Use of Drugs; 4 Hematology Oncology and Stem Cell Transplantation Research Center, Department of Hematology-Oncology, Shariati Hospital; 5 Department of Epidemiology and Biostatistics, School of Public Health; 6 Faculty of Pharmacy, Tehran University of Medical Sciences, Tehran, Iran

**Keywords:** Thalassemia, Hematopoietic Stem Cell Transplantation, Vitamin A, Vitamin E, Dietary Supplements

## Abstract

***Objective:*** Thalassemia along with hematopoietic stem cell transplantation (HSCT) can lead to major oxidative stress. Vitamins A and E are antioxidants which protect membrane from lipid peroxidation. We sought to determine for the first time, whether vitamins A and E supplementation is efficacious in maintaining or increasing plasma level of these vitamins in thalassemic children undergoing HSCT.

***Methods:*** A cross-sectional study was performed on 50 children with β-thalassemia major hospitalized for HSCT. Patients took a daily multivitamin. Plasma vitamins A and E levels were measured at four different times: on admission, HSCT day (day 0), day 7 and day 14 after HSCT.

***Findings*** ***:*** Plasma vitamin A and E were abnormal on admission in most patients (62.0% and 60.0% respectively). Ratio of patient with normal to abnormal plasma level of the vitamins improved from baseline to a peak on day 7 then deteriorated afterward until day 14. There was an increasingly positive correlation between daily oral intake and plasma vitamin A at different times, but plasma vitamin E showed inverse correlation at first which tended towards no correlation subsequently. In multivariate analysis, supplementation significantly changed plasma level of vitamin A at different measurement time (*P*=0.001) within study subjects. But, plasma level of vitamin E showed no significant difference (*P*=0.2).

***Conclusion:*** Our findings suggest that oral supplementation could have beneficial effects due to increasing plasma vitamin A level and preventing plasma vitamin E depletion.

## Introduction

Thalassemia is the most common congenital disease in the world^[^^1^^]^, and thalassemia major is a severe and usually fatal kind of anemia^[^^2^^]^. Morphologic abnormality of erythrocytes in thalassemia exposes them to superior oxidative stress caused by globulin chains auto-oxidation, iron overload, low level of adult hemoglobin (HbA)^[^^3^^]^, and lipid peroxidation^[^^4^^]^. Hematopoietic stem cell transplantation (HSCT) is the only available curative approach to thalassemia^[^^4^^,^^5^^]^. HSCT is an aggressive therapeutic procedure which includes administration of high dose conditioning regimen (CR) followed by precursor hematopoietic stem cell infusion^[^^2^^]^, with or without total body irradiation (TBI)^[^^6^^]^. HSCT is associated with the formation of large quantity of reactive oxygen/nitrogen species^[^^6^^-^^8^^]^ mediated by free radicals forming oxidative products^[^^9^^]^. Therefore, thalassemic patients undergoing HSCT will certainly encounter a major and maybe cumulative oxidative stress condition which depletes critical plasma and tissue antioxidants^[^^6^^]^. Vitamin E is a lipid-soluble proxyl radical scavenger^[^^10^^,^^11^^]^. It is the major antioxidant protecting membrane lipid against peroxidative damages in plasma and erythrocytes^[^^12^^]^. Vitamin A is also an effective lipid soluble^[^^12^^,^^13^^]^ oxygen quencher antioxidant and inhibits free radical reactions as well^[^^12^^]^.

 A number of studies have assessed level of vitamin A and E in thalassemic patients^[^^1^^,^^3^^,^^14^^,^^15^^]^. A few have found a very low level of serum vitamin E^[^^14^^]^ and a dramatic reduction in plasma vitamin A levels^[^^15^^]^. In two studies of vitamin A and E as supplementation, researchers observed considerable reduction in lipid peroxidation of erythrocyte membranes as outcome^[^^1^^,^^3^^]^. In addition, some studies on HSCT patients have also reported a decrease in serum concentration of some nutrient antioxidants such as α-tocopherol and β-carotene^[^^7^^,^^16^^-^^18^^]^. Livrea et al showed that administration of antioxidants by supplementation of vitamin A and E could be advisable and beneficial in thalassemia^[^^15^^]^. Moreover, Durkens et al found that supplementation of antioxidants in HSCT patients could be effective^[^^9^^]^. Sabuncuoglu et al also claimed that vitamin supplementation was sufficient to maintain α-tocopherol and β-carotene plasma levels after CR in children undergoing HSCT^[^^19^^]^. So, it can be expected that the administration of these supplements in thalassemic patients undergoing HSCT will also be advantageous.

 As far as we know, there has been no research on vitamin A and E supplementation in this specific population, i.e. children with β-thalassemia major undergoing curative HSCT. We aimed to find out further evidence on the effects of supplementation on maintaining adequate plasma levels of vitamin A and E to provide a rational basis for vitamin A and E administration.

## Subjects and Methods

**Study design and setting**

We carried out a prospective cross-sectional observational study in the Hematology-Oncology and Bone Marrow Transplantation Research Center at Shariati Hospital, Tehran, from July 2009 to June 2011. Our institutional review board approved the study proposal. The study was approved by the Committee for Research Ethics and followed the Declaration of Helsinki.

**Participants**

Children aged 7.973.54 years with β-thalassemia major hospitalized for allograft HSCT in pediatric ward were recruited for this study. All patients’ guardians signed an informed consent before the study. 

**Conditioning Regimen**

All patients received CR for allogenic HSCT. Their conditioning regimen consisted of Busulfan (3.5mg/kg for 4 days) and Cyclophosphamid (40 mg/kg in thalassemia major class III and 50 mg/kg in thalassemia major class I and II for 4 days after Busulfan). Anti-Thymocyte Globulin (rabbit ATG, 1.25mg/kg for 2 days before HSCT) was added for patients who received peripheral blood progenitor cells or with matched unrelated donors. 

**Supplementation**

All 50 patients had the same nutritional intake based on the similar daily diet in the unit, and they all received food and medicine by mouth during the study and no patient went under total parenteral nutrition (TPN). All patients took a daily multivitamin (multi-Sanostol, Nycomed, Germany), started at the beginning of patients’ hospitalization after collecting baseline sample and it continued throughout their hospitalization, which was a part of institutional protocol. They received 40ml/day (containing 9600 IU vitamin A and 8 mg vitamin E), if their weight was more than 20 Kg; and 20 ml/day (4800 IU vitamin A and 4 mg vitamin E), if they were less than 20 Kg, both in two divided doses. 

**Outcome measures**

 The study main outcomes were changes in vitamin A and E serum concentration levels; therefore we could evaluate the effect of multivitamin administration in prevention of vitamin A and E concentration depletion in these patients.

 We also categorized vitamin A levels of less than 0.2 µg/ml and vitamin E levels of less than 5.0 µg/ml as “abnormal”, according to the laboratory normal range which was considered as the standard, and compared the normal to abnormal ratios.

 We gathered baseline and clinical data during one month of observation. We measured plasma vitamin A and vitamin E levels at four different times: on admission (baseline), on HSCT day (day 0), 1 week after HSCT (day 7), and 2 weeks after HSCT (day 14).

**Laboratory measurement of plasma levels of vitamins A and E**

 Four Blood samples (5ml) were collected from central venous catheters into a citrated blood collection tubes, then they were centrifuged for 10 minutes at 1000×g. The plasma was separated in cryogenic vials then stored at -70 C till preparation for HPLC analysis. Final steps were performed in a semi dark room, away from the direct light. 150 μl internal standard (ethanolic retinol acetate 50 μg/ml) was mixed with 150 μl plasma by vortex-mixer for 30 seconds. Then 300 μl hexane was added to the mixture and mixed again by vortex-mixer for 2 minutes. The Mixture was centrifuged at 6000×g for 5 minutes. 150 μl of the hexane layer (upper layer) picked out, and then the solvent was purged under nitrogen gas flow. Afterward 150 μl of mobile phase (95% methanol and 5% butanol) was added to vials, the transparent solution was ready for HPLC injection. All the procedure and sample storing were performed under light protected condition. Thirty minutes before sample injection the mobile phase was passed through the column. Twenty μl of samples were injected to HPLC column (250 4.6mm) Pontosil C18; Knauer Inc. by auto sampler and Eurochrom 2000 software. Dual ultraviolet-visual light detection (sensitivity 5 10¯) was used at the flow rate 5ml/min. Absorbance at 20 nm and 292 nm specified concentration of vitamin A and E respectively.

**Biases**

We included data from all thalassemic patients admitted for HSCT during the study period with no exclusion. Based on expert opinion, the case mix of admissions in our study time was comparable to other periods. 

 Standardized measurement protocols and calibration methods were developed and tested in order to minimize possible measurement errors.

**Statistical analysis**

Descriptive statistics were shown by frequencies and percentages for categorical variables and by mean+/- standard error for quantitative variables. For analysis of categorical variables, we used Chi-square test and for quantitative variables Student t-test and Mann-Whitney-U test.

 For comparison of vitamin E and A levels in different times of HSCT within subjects (i.e. baseline, day 0, day 7 and day 14), we conducted repeated measure analysis of variance (ANOVA) with pair-wise comparisons. Pearson correlation coefficient was calculated to compare levels of vitamin A and E levels at different two-by-two times. A *P*-value of less than 0.05 was considered significant. All statistical analysis was done using Statistical Packages for Social Sciences (SPSS) software version 16.0 (formerly SPSS Inc., USA, Chicago), for statistical analysis. 

## Findings

We gathered data from 50 patients. No patient died or was lost during follow-up.

**Fig. 1:**
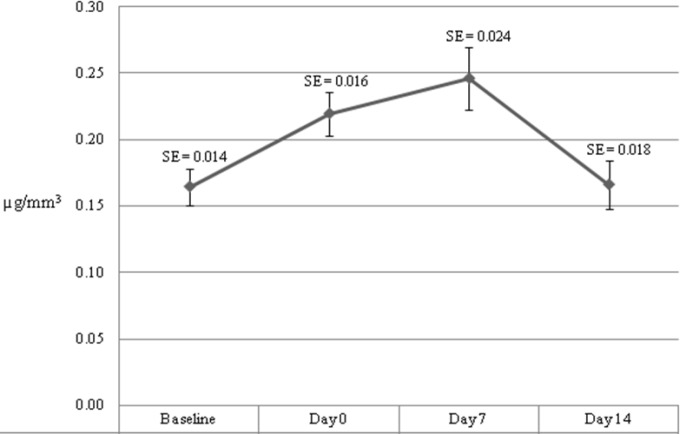
Mean (±standard error) plasma level of vitamin A at different times (µg/mm^3^)

**Fig. 2:**
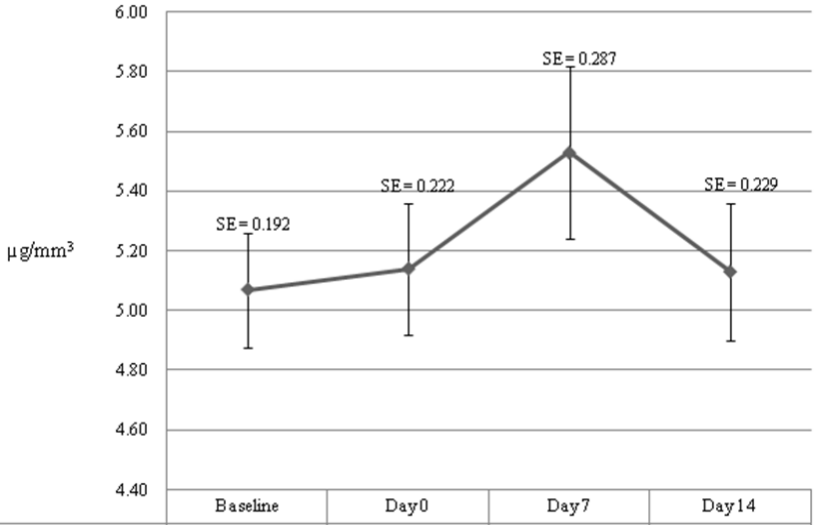
Mean (±standard error) plasma level of vitamin E at different times (µg/mm^3^)

 Baseline and clinical data are summarized in Table 1. We calculated mean and standard error for plasma levels of vitamin A and E at differenttimes. Figs. 1 and 2 show trends of changes in plasma level of vitamin A and E. For both vitamins, we observed an increase from baseline to a peak on day 7; but, a decreasing trend was seen after day 7 until day 14. Plasma level of vitamin A was significantly different at different times of the study (*P*=0.001) within study subjects. But, plasma level of vitamin E showed no significant difference (*P*=0.2) (Table 2). There was an increasingly positive correlation between daily vitamin A oral intake and plasma level of vitamin A at different times. Daily vitamin E oral intake and plasma level of vitamin E showed inverse correlation at first which tended towards no correlation at subsequent measurement times (Table 3).

**Table 1:** Plasma levels of vitamins: baseline, clinical and follow-up Data

**Variable**		**n (%)**	**Mean**	**SD**
**Baseline Data**	Age (years)	50 (100.0)	7.98	3.54
	Gender Male	26 (52.0)		
	Female	24 (48.0)		
	Body Mass Index (kg/m2)	50 (100.0)	14.98	1.59
**Clinical Data – Before HSCT** **Type of Thalassemia**	Class 1	14 (28.0)		
	Class 2	20 (40.0)		
	Class 3	14 (28.0)		
	Sickle Thalassemia	2 (4.0)		
**Source of Allograft Transplantation**	Peripheral Blood	29 (58.0)		
	Bone Marrow	21 (42.0)		
**Donor Type**	Sibling	42 (84)		
	Unrelated	8 (16)		
	Ferritin status (ng/Lit)	50 (100.0)	2221.9	1785.6
	Transfusion Start Time (months)	46 (92.0)	17.89	
	Chelation Therapy Start Time (mo)	44 (88.0)	37.09	
**Clinical Data – Conditioning regimen**	Bu, En	13 (26.0)		
	Bu, En, ATG	37 (74.0)		
**Clinical Data – Oral Supplementation**	Vitamin A (IU/d)	50 (100.0)	2500.8	866.1
	Vitamin E (mg/d)	50 (100.0)	2.1	0.7
**Clinical Data – After HSCT**	WBC engraftment time (days)	50 (100.0)	13.1	4.0

SD: Standard Deviation; HSCT: hematopoietic stem cell transplantation; Bu: Busulfan; En: Endoxan; ATG: Anti-Thymocyte Globulin

Ratio of normal to abnormal for plasma level of vitamin A, likewise vitamin E, increased from baseline to a peak on day 7; but, a decreasing ratio was seen after day 7 until day 14 (Table 4).

## Discussion

Thalassemic patients undergoing HSCT, suffer from low plasma levels of vitamin A and E. In our study, plasma levels of vitamin A and E in most of our patients were abnormal at baseline (62.0% and 60.0% respectively). It seems that oral vitamin supplementation could have beneficial effects to maintain adequate plasma levels of these vitamins for this population at least in short-term use. It increased plasma vitamin A level and prevented plasma vitamin E depletion during admission for HSCT.

**Studies on thalassemia**

In the present study, we observed vitamin A and E deficiency at baseline, similar to other studies. 

**Table 2:** Within subject difference of plasma levels of vitamin A and vitamin E at different times using repeated measure ANOVA

**Effect**		**Value**	**F (Exact statistic)**	**Error df**	**Sig**
**Vitamin A**	Wilks' Lambda	.691	7.011	47.000	.001
**Vitamin E**	Wilks' Lambda	.915	1.450	47.000	.240

Livrea et al observed remarkable reduction of β-carotene and α-tocopherol in thalassemic patients^[^^15^^]^. Decline or depletion of these vitamins can be explained by impairment of liver function and preoxidative process. Chronic hepatic iron overload causes a significant serum lipid reduction which can lead to simultaneous reduction in serum vitamin A and E^[^^20^^,^^21^^]^. Therefore we have a considerable hyperconsumption of vitamin E^[^^15^^,^^20^^]^ as a radical scavenger^[^^21^^]^ and possibly other lipid soluble antioxidants for neutralizing oxidative processes, both at liver ^[^^15^^, ^^21^^]^ and membrane level^[^^14^^]^.

 Unlike our results, in one study no significant variation of vitamin A was observed^[^^21^^]^. One possible explanation is that severe liver damage might interfere with hepatic uptake or metabolism of β-carotene, which will cause a paradoxical relative elevation of β-carotene in serum of these patients^[^^15^^]^.

 Das et al trial on thalassemic children using vitamin E supplementation for 4 weeks, reported no significant change in vitamin E serum level; but, as they took only one sample on the 29^th^ day, they would not have been able to show any trend in vitamin E concentration during their 4-week follow-up. They discussed that gastrointestinal tract abnormality in absorption and ongoing consumption of a great amount of vitamin E for scavenging reactive mediator formed from oxidative stress may lead to observed results^[^^2^^]^. The former reason was later debated by Rachmilewitz et al^[^^20^^]^. In Rachmilewitz et al study, patients received a daily amount of 1050 IU vitamin E in a 16-month period. The authors reported almost four-fold increase in serum level of vitamin E after 14 months. Trends for serum level of vitamin E were different and opposite in some cases and the reason was not clear^[^^20^^]^. In comparison, the probable reasons that we did not see significant changes in vitamin E in our patients despite the HSCT procedure, might be the shorter time and lower oral dose of vitamin E supplementation regimen in our study.

**Studies on HSCT**

Two studies on patients undergoing HSCT reported that serum level of vitamin A and E was normal at baseline before CR^[^^17^^,^^18^^]^. The difference to our finding at the admission day could be due to difference in target populations as our study is focused solely on thalassemic patients.

 High et al^[^^17^^]^ assessed changes of vitamin A and E at 4 different times similar to our study. They report significant reduction of vitamin A from normal concentration at baseline until day 7. But our study showed a significant increase in vitamin A level until day 7 as a result of supplementation. They also found notable serum level of vitamin E depletion. But we did not observe any reduction in plasma level of vitamin E as supplementation result. They also discussed that plasma vitamin A and E spontaneously recover by day 14^[^^17^^]^. Probably because of patients’ underlying disease which resulted in vitamin deficiency at baseline^[^^1^^,^^3^^,^^10^^,^^14^^,^^15^^]^, plasma level of vitamins would not reach completely normal values even after three weeks supplementation.

**Table 3:** Correlation of daily vitamin A and E oral intake and plasma level of vitamin A and E at different times

**Corresponding plasma level of vitamin**		**Baseline**	**Day 0**	**Day 7**	**Day 14**
**Daily Vitamin A Oral Intake**	Pearson Correlation Coefficient	0.101	0.275	0.378	0.389
	*P. *value	0.485	0.053	0.007	0.005
**Daily Vitamin E Oral Intake**	Pearson Correlation Coefficient	-0.339	-0.182	0.017	-0.020
	*P. *value	0.016	0.207	0.909	0.889

**Table 4:** Ratio of normal to abnormal for plasma level of vitamin A and E at different times

**Variable**	**Time**	**Normal**	**Abnormal**	**Ratio**
**Vitamin A**	Baseline	19 (38.0%)	31 (62.0%)	0.61
	Day 0	19 (38.0%)	31 (62.0%)	0.61
	Day 7	30 (60.0%)	20 (40.0%)	1.50
	Day 14	25 (50.0%)	25 (50.0%)	1.00
**Vitamin E**	Baseline	20 (40.0%)	30 (60.0%)	0.67
	Day 0	25 (50.0%)	25 (50.0%)	1.00
	Day 7	27 (54.0%)	23 (46.0%)	1.17
	Day 14	18 (36.0%)	32 (64.0%)	0.56

 According to Goncalves et al, vitamin A and E decline on day 14 could be the result of increasing lipid peroxidation on day 10 as late effect of BuCy regimen^[^^6^^]^. Another possible cause could be vitamin status variation caused by poor intake in HSCT patients due to malabsorption^[^^22^^,^^23^^]^, anorexia, nausea, vomiting and mucositis^[^^23^^]^. Chemotherapy-induced mucosal injury usually develops around day 7 and achieves its peak in two weeks (day 14)^[^^24^^]^. 

 Sabuncuoglu et al observed a significant increase in vitamin E plasma level due to supplementation in HSCT patients. In comparison vitamin E supplementation dose was higher than in our study^[^^19^^]^. 

 A few studies assessed total parenteral nutrition (TPN) in patients undergoing HSCT. They reported normal serum levels of vitamin A and E at baseline^[^^7^^,^^18^^]^. Clemens et al showed a significant reduction in α-tocopherol and β-carotene during conditioning. Moreover, vitamin E supplementation and vitamin A and E dosages were not sufficient to maintain appropriate vitamin A and E status^[^^18^^]^. In contrast, our study showed a significant increase for vitamin A and a steady-state level of vitamin E during conditioning. This variation could be due to different age structure and fewer sample population in the study by Clemens et al. In another study by Jonas et al on 40 adult patients undergoing HSCT, changes in vitamin E level from baseline to day 14 was close to our results; they first faced an increase and then a reduction in serum level of vitamin E^[^^7^^]^.

 We did not examine the level of oxidative stress factors such as lipid peroxidation of RBC in our study. Therefore the positive effects of supplementation on plasma levels of vitamins A and E on oxidative stress reduction is not known.

## Conclusion

We found beneficial effects of vitamin A and E administration in thalassemic patients undergoing HSCT in order to prevent vitamin depletion. We recommend further studies to investigate the natural variation in plasma level of vitamin A and E without any supplementation, and thereafter, at least a well-designed clinical trial with longer follow-up period.
